# Differential Cellular Balance of Olfactory and Vomeronasal Epithelia in a Transgenic BACHD Rat Model of Huntington’s Disease

**DOI:** 10.3390/ijms23147625

**Published:** 2022-07-10

**Authors:** Lina-Marielle Krysewski, Nicole Power Guerra, Annika Glatzel, Carsten Holzmann, Veronica Antipova, Oliver Schmitt, Libo Yu-Taeger, Huu Phuc Nguyen, Andreas Wree, Martin Witt

**Affiliations:** 1 Department of Anatomy, Rostock University Medical Centre, 18057 Rostock, Germany; linamarielle@hotmail.com (L.-M.K.); nicole.powerguerra@uniklinikum-dresden.de (N.P.G.); annika.glatzel@sf.mpg.de (A.G.); veronica.antipova@medunigraz.at (V.A.); oliver.schmitt@medicalschool-hamburg.de (O.S.); andreas.wree@med.uni-rostock.de (A.W.); 2 Rudolf-Zenker-Institute for Experimental Surgery, Rostock University Medical Centre, 18057 Rostock, Germany; 3 Smell and Taste Clinic, Department of Otorhinolaryngology, TU Dresden, 01034 Dresden, Germany; 4 Institute of Medical Genetics, Rostock University Medical Centre, 18057 Rostock, Germany; carsten.holzmann@med.uni-rostock.de; 5 Gottfried Schatz Research Center for Cell Signaling, Metabolism and Aging, Macroscopic and Clinical Anatomy, Medical University of Graz, 8010 Graz, Austria; 6 Department of Anatomy, Medical School Hamburg, University of Applied Sciences and Medical University, 20457 Hamburg, Germany; 7 Department of Human Genetics, Ruhr University Bochum, 44780 Bochum, Germany; libo.yu-taeger@med.uni-tuebingen.de (L.Y.-T.); huu.nguyen-r7w@ruhr-uni-bochum.de (H.P.N.)

**Keywords:** vomeronasal organ, main olfactory epithelium, Huntington’s disease, transgenic rat model, olfaction, buried pellet test

## Abstract

*Background.* For neurodegenerative diseases such as Huntington’s disease (HD), early diagnosis is essential to treat patients and delay symptoms. Impaired olfaction, as observed as an early symptom in Parkinson´s disease, may also constitute a key symptom in HD. However, there are few reports on olfactory deficits in HD. Therefore, we aimed to investigate, in a transgenic rat model of HD: (1) whether general olfactory impairment exists and (2) whether there are disease-specific dynamics of olfactory dysfunction when the vomeronasal (VNE) and main olfactory epithelium (MOE) are compared. *Methods.* We used male rats of transgenic line 22 (TG22) of the bacterial artificial chromosome Huntington disease model (BACHD), aged 3 days or 6 months. Cell proliferation, apoptosis and macrophage activity were examined with immunohistochemistry in the VNE and MOE. *Results.* No differences were observed in cellular parameters in the VNE between the groups. However, the MOE of the 6-month-old HD animals showed a significantly increased number of mature olfactory receptor neurons. Other cellular parameters were not affected. *Conclusions.* The results obtained in the TG22 line suggest a relative stability in the VNE, whereas the MOE seems at least temporarily affected.

## 1. Introduction

Huntington’s disease (HD) is an inherited, incurable, neurodegenerative and also multi-system disorder associated with progressive movement, psychiatric and cognitive symptoms manifesting between 30 and 40 years of age [1,2]. The pathology arises from the mutation in the gene for huntingtin, increasing the number of repeats of CAG base triplets [3,4]. The majority of HD patients possesses between 40 and 55 CAG triplet repeat expansion, leading to an unfolded abnormal huntingtin protein and, ultimately, to neuronal dysfunction and cell death [5]. Using neuroimaging techniques, Tabrizi et al. [6] demonstrated that, in addition to atrophies of the caudate nucleus and putamen [7], generalized atrophy of the brain is present in HD, with massive striatal cell death of GABAergic spinal projection neurons [8,9]. Prior to a motor diagnosis, HD patients develop subtle signs of Huntington’s disease years beforehand [5]. Olfactory dysfunction as a possible early symptom of neurodegenerative diseases has been shown previously in Parkinson’s disease, Alzheimer’s disease and Niemann-Pick disease type C1 [10,11,12,13,14]. In HD, olfactory impairment prior to verbal and visual disabilities has been observed by Moberg et al. (1987) [15] and Nordin et al. (1995) [16]. In addition, Bacon Moore et al. (1999) discovered odor sensitivity and odor memory deficits in HD patients [17]. In mouse studies, Lazic et al. (2007) observed reduced cell migration and reduced plasticity in the piriform cortex [18]. This observation was complemented with findings in a knock-in mouse model, for which Menalled et al. (2003) found increased levels of dopamine [19], which is, though enigmatic, associated with olfactory disturbances in neurodegenerative diseases [20]. In the same period, von Hörsten et al. (2003) developed the first rat model, which best represents the most common adult, late-manifesting and slowly progressive form of HD [21]. This bacterial artificial chromosome Huntington disease (BACHD) rat model was further developed by Yu-Taeger et al. (2012) [22]. Using this BACHD model, Lessard-Beaudoin et al. (2019) demonstrated atrophy of the olfactory bulb with an associated activation of caspase-3 (for apoptosis) in rats of the transgenic lines (TG) 5 and 9 [23]. The atrophy in the olfactory bulb of the HD rats was restricted to the internal layers [23]. Structural abnormalities were additionally observed in the piriform and entorhinal cortices in a mouse model of HD [24]. The BACHD rat models established by Yu-Taeger et al. (2012) revealed several rat lines with classical neuropathological and behavioral signs of HD [22]. We used one of these rat lines (TG22) to study olfactory parameters that may give possible clues for chemosensory deficits in HD. In contrast to the TG5 and TG9 lines, no olfactory-related data are available for the new TG22 line.

Of particular interest in the chemosensory systems are the olfactory receptor neurons (ORNs), which have the capacity for continuous neurogenesis [25]. Olfactory neurogenesis is a meticulously controlled cascade of molecular events resulting in balanced proliferation and differentiation of basal and progenitor cells in the olfactory mucosa [25,26,27]. ORNs are located in the olfactory epithelium and constitute the vomeronasal organ (VNO) and the main olfactory epithelium (MOE) [28]. We previously described the influence of Niemann-Pick type C1 (NPC1) mutation in a mouse model for the MOE [11] and vomeronasal epithelium (VNE) [27] and showed that vomeronasal receptor neurons, unlike ORNs, are less sensitive to NPC1 pathology.

Referring to our aforementioned NPC1 study, the present work investigated whether the described olfactory phenotype is also present in the MOE and VNE of the TG22-BACHD rats of Yu-Taeger et al. (2012) with a concurrent reduced regenerative potential in the MOE [22]. Furthermore, we evaluated differences in the MOE and VNE regarding cell proliferation, cell death and the integrity of olfactory receptor neurons in young rats (3 days old) and adolescent rats (aged 6 months).

## 2. Results

### 2.1. Validation of the Bacterial Artificial Chromosome Huntington Disease (BACHD) Rat Model

**Figure 1:**
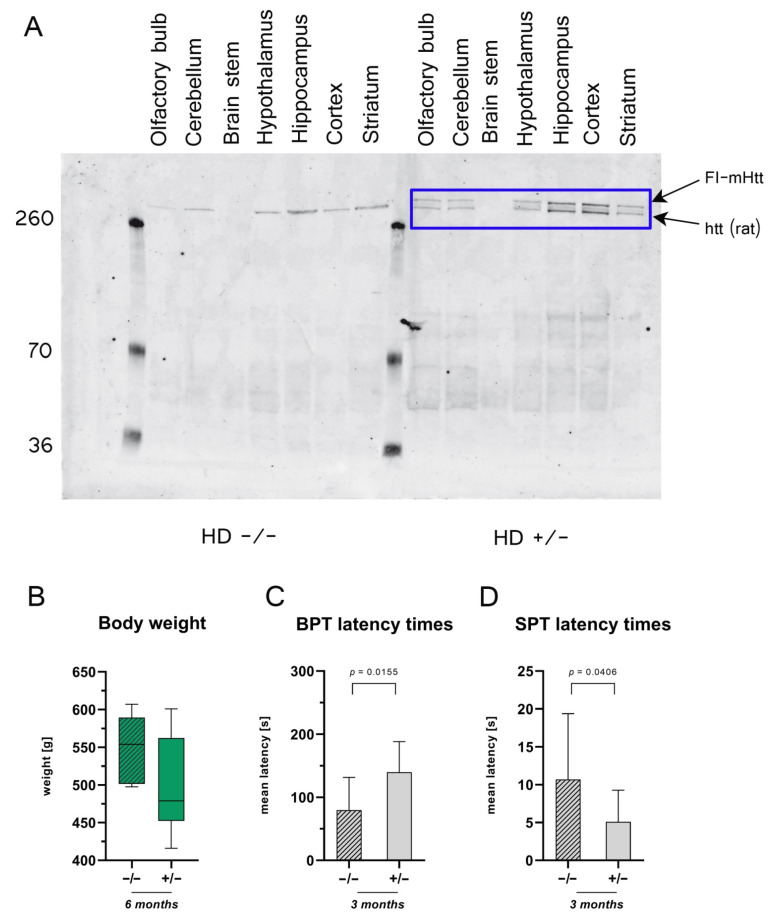
Validation of the bacterial artificial chromosome Huntington disease (BACHD) rat model. (**A**) Western blot analysis of mHTT expression in various brain regions in transgenic line 22 (TG22) using antibody MAB2166. mHTT was present in the olfactory bulb, cerebellum, hypothalamus, hippocampus, cortex and striatum but lacking in the brain stem (blue rectangle). The mHTT expression correlated with the expression of endogenous htt. (**B**) Body weights of 6-month-old (6 M) rats; *n* = 6, respectively. (**C**) Mean latency times for 3-months-old rats in the buried pellet test (BPT), *n* = 10, respectively. Latency times for finding a buried pellet were averaged over 5 days. The significance of differences between groups was tested with an unpaired t-test ((**B**) *F* value = 2.335, degree of freedom = 5; (**C**) *F* value = 1.146, degree of freedom = 9). Data are presented as means ± SD and statistical significance was set at *p* < 0.05. (**D**) Mean latency times in the surface pellet test (SPT) for 3-months-old rats, *n* = 10, respectively. The significance of differences between groups was tested with an unpaired *t*-test ((**B**) *F* value = 2.335, degree of freedom = 5; (**C**) *F* value = 1.146, degree of freedom = 9) or with a Mann–Whitney test (**D**). Data are presented as means ± SD and statistical significance was set at *p* < 0.05.

First, we used Western blot analysis to verify the insertion of bacterial artificial chromosome mutant huntingtin protein (*mHTT*). The blue box in Figure 1A indicates the abundance of the mutant Huntingtin protein inserted in BACHD rats but not in the wildtype (WT) littermates. Body weights within 6 months were genotype-independent and potential gains were not significantly different (Figure 1B).

### 2.2. Three-Month-Old BACHD Rats Have Olfactory Deficits

A cross-sectional buried pellet test (BPT) was performed on rats aged 3 months. *HD^+/−^* rats exhibited higher latency times when compared to their WT littermates (Figure 1C), indicating impairment of olfactory detection ability. The latencies in the surface pellet test (SPT), however, were significantly reduced in *HD^+/−^* rats (Figure 1D). In habituation/learning assessment over 5 days, the transgenic rats showed no differences in latency times when compared to their wildtype littermates (Figure S1). The purpose of the BPT is to check the olfactory perception of animals (Figure 1C). However, the function of the surface pellet test is to rule out motor deterioration, visual impairment and altered food motivation. Due to the very short latency of the surface pellet test (SPT), it can be assumed that the differences were due to scatter in the measured data and the fact that the SPT was only performed once (Figure 1C). In summary, the comparison of the data of the BPT and the SPT speaks for clear damage to the odor perception of the BACHD rats, which was obviously not superimposed by motor and/or visual deteriorations.

### 2.3. Morphology, Cell Proliferation and Apoptosis Are Independent of Genotype

**Figure 2:**
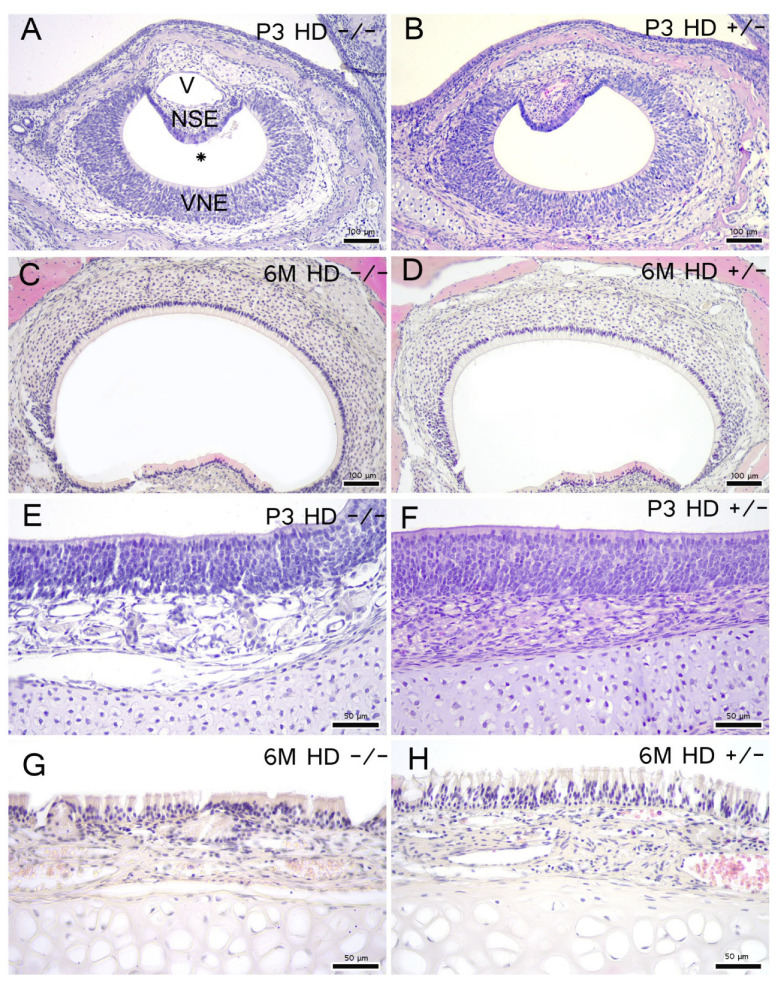
Overview of vomeronasal and main olfactory epithelia morphology: H&E staining of 3-day-old (P3) and 6-month-old (6 M) vomeronasal epithelium (**A**–**D**) and main olfactory epithelium (**E**–**H**). The semilunar-shaped VNO consists of a vomeronasal duct (*), sensory vomeronasal epithelium (VNE), non-sensory epithelium (NSE), vomeronasal cartilage (Ca), vomeronasal nerve and glands (not indicated). V = vein. After Witt and Woźniak [29]. Scale bars: (**A**–**D**) 100 µm; (**E**–**H**) 50 µm.

Subsequently, the HD genotype showed no effect on the morphology of the semilunar-shaped vomeronasal epithelium (VNE; Figure 2A–D) or the main olfactory epithelium (Figure 2E–H), nor were signs of apoptosis or necrosis visible.

**Figure 3:**
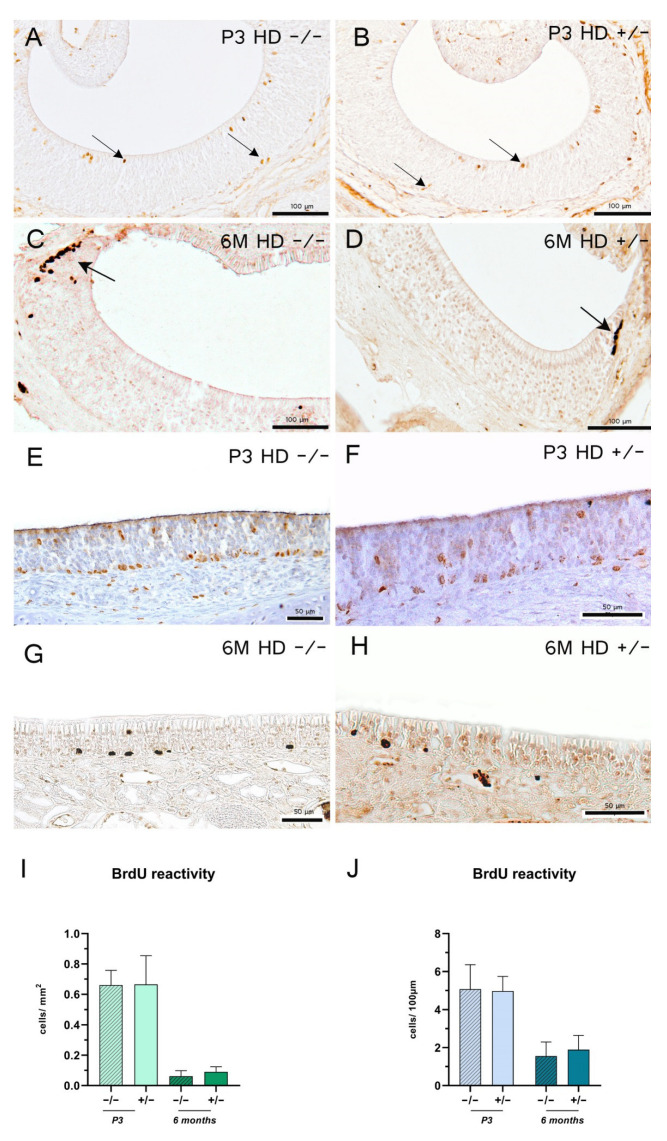
5-Bromo-2′-deoxy-uridine (BrdU) detection of 3-day-old (P3) and 6-month-old (6 M) rats 2 h post-injection. (**A**–**H**) Representative immunohistochemical reaction of BrdU-positive cells in the vomeronasal epithelium (**A**–**D**) and main olfactory epithelium (**E**–**H**); arrows indicate BrdU-positive cells. Scale bars: (**A**–**D**) 100 µm; (**E**–**H**) 50 µm. Column bars show BrdU reactive cell counts per mm^2^ (**I**) or per 100 µm (**J**); *n* = 6; (**J**) +/− 6 M: *n* = 5. The significance of differences between groups was tested with mixed-effects analysis followed by Bonferroni’s multiple comparisons (age x genotype interaction: (**I**) *F* value = 0.07894, degree of freedom = 1; (**J**) *F* value = 1.785, degree of freedom = 1. Data are presented as means ± SD and statistical significance was set at *p* < 0.05.

To investigate cell proliferation and apoptosis, 5-bromo-2′-deoxy-uridine (BrdU) and activated caspase-3 reactions were performed [30,31,32]. Two hours before euthanasia, rats were i.p. injected with 0.05 mg BrdU per gram of body weight. The integration of the thymidine analogue during the S-phase of the DNA synthesis can be visualized immunhistochemically with BrdU antibodies. While the 3-day-old rats exhibited a homogeneous distribution of BrdU-positive cells in the basal and apical compartments throughout the VNE (Figure 3A,B), the adult rats showed a conspicuous accumulation in the lateral compartment—the proliferation zone [27]—with a horizontal migration (Figure 3C,D). In the MOE, a higher abundance of BrdU-positive cells was visible in the basal cells of the epithelium with an apical cell migration (Figure 3E–H). The transgenic rats did not show any decrease in cell proliferation in the VNE or in the MOE (Figure 3E,J). However, cell apoptosis was not influenced by the genotype in the 6-month-old rats in either epithelia (Table 1).

### 2.4. Transgenic Rats Revealed an Increase in Mature Olfactory Receptor Neurons in the Main Olfactory Epithelium

**Figure 4:**
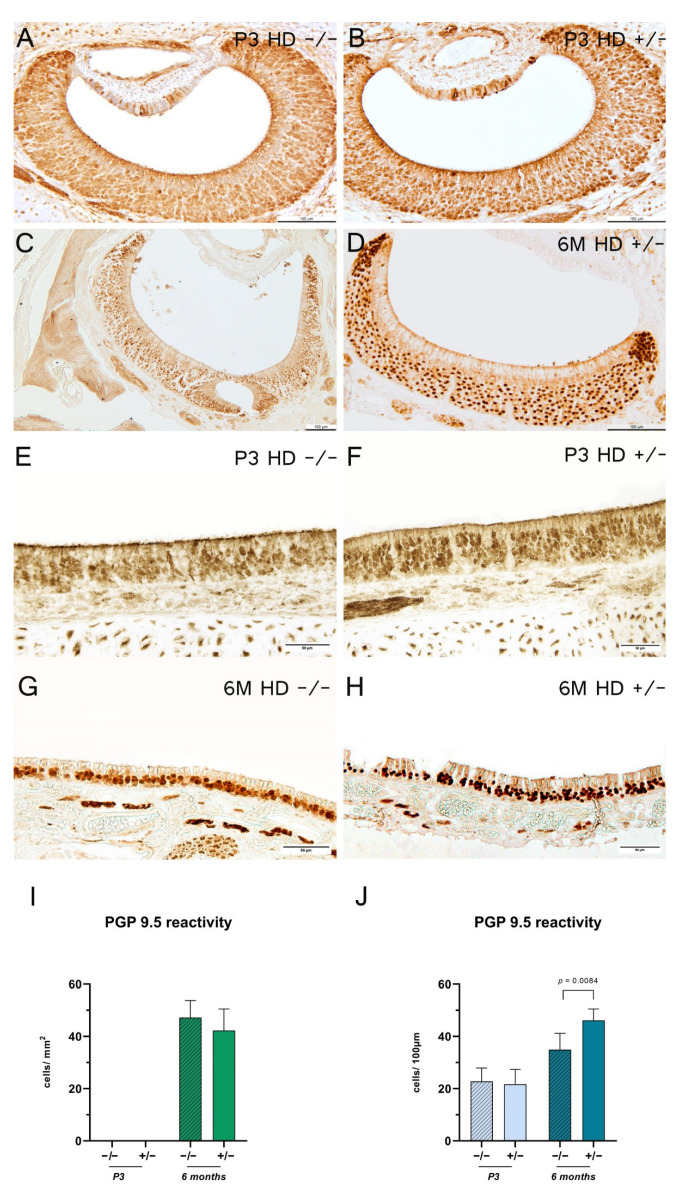
Protein gene product (PGP) 9.5 reaction for 3-day-old (P3) and 6-month-old (6 M) rats. (**A**–**H**) Representative immunohistochemical reaction of PGP 9.5-positive cells in the vomeronasal epithelium (**A**–**D**) and main olfactory epithelium (**E**–**H**). Scale bars: (**A**–**D**) 100 µm; (**E**–**H**) 50 µm. Column bars show PGP 9.5-reactive cell counts per mm^2^ (**I**) or per 100 µm (**J**); (**I**) P3 and 6 M: *n* = 5–6; (**J**) P3: *n* = 6, 6 M: *n* = 5. The significance of differences between groups was tested with an unpaired t-test ((**I**) *F* value = 1.601, degree of freedom = 4) or with mixed-effects analysis followed by Bonferroni’s multiple comparisons (age x genotype interaction: (**J**) *F* value = 7.055, degree of freedom = 1). Data are presented as means ± SD and statistical significance was set at *p* < 0.05.

**Figure 5:**
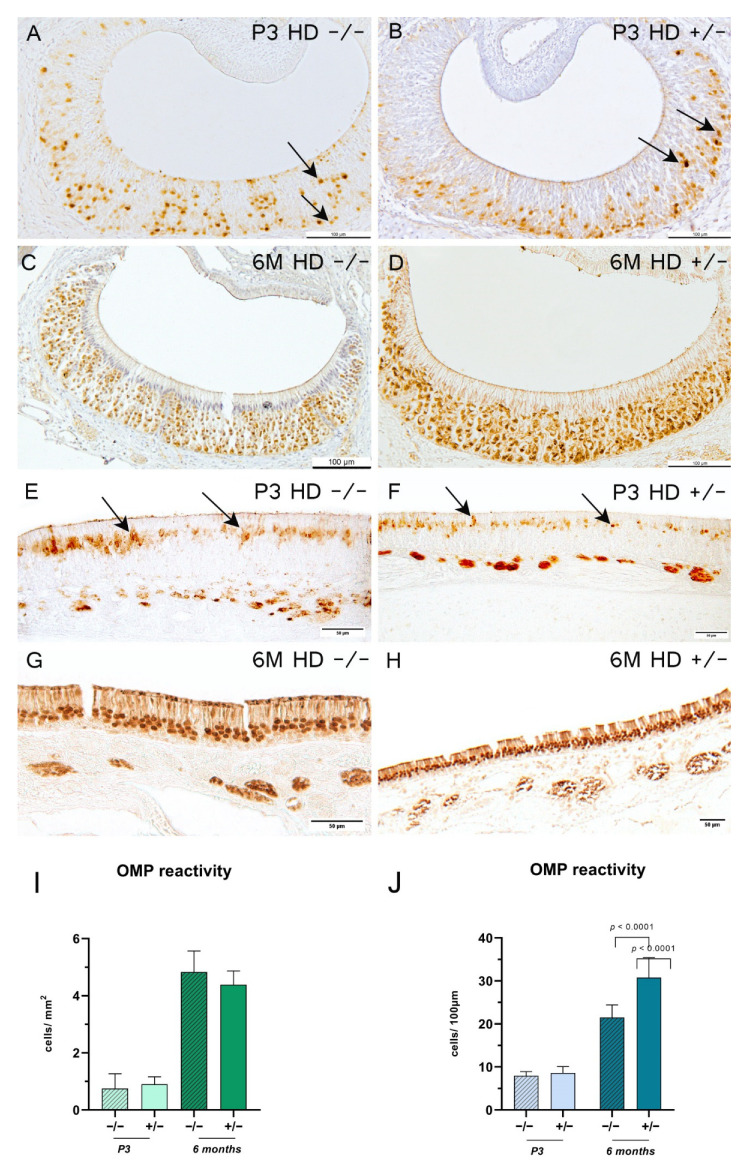
Olfactory marker protein (OMP) reaction for 3-day-old (P3) and 6-month-old (6 M) rats. (**A**–**H**) Representative immunohistochemical reaction of OMP-positive cells in the vomeronasal epithelium (**A**–**D**) and main olfactory epithelium (**E**–**H**). Arrows indicate OMP-positive cells. Scale bars: (**A**–**D**) 100 µm; (**E**–**H**) 50 µm. Column bars show OMP-reactive cell counts per mm^2^ (**I**) or per 100 µm (**J**); *n* = 6; (**J**) 6 M: *n* = 5. The significance of differences between groups was tested with mixed-effects analysis followed by Bonferroni’s multiple comparisons (age x genotype interaction: (**I**) *F* value = 1.915, degree of freedom = 1; (**J**) *F* value = 15.28, degree of freedom = 1). Data are presented as means ± SD and statistical significance was set at *p* < 0.05.

Protein gene product (PGP) 9.5-positive cells represent the total number of neurons at different levels of maturity [33]. The VNE of 3-day-old rats showed a PGP 9.5-positive reaction with no definite delineation, which precluded a quanti fication of the cells (Figure 4A,B). However, increased reaction of the dendrites to the lumen was observable. The VNE in the 6-month-old rats showed a shift of reactive cells from rostral to occipital, with a more homogeneous distribution of the abundance of proliferating receptor neurons in both compartments (Figure 4C,D). This effect, however, was genotype-independent. In contrast, the MOE showed a more homogeneous distribution of positive-reacted receptor neurons in both age groups, with a significant increase of proliferating neurons in *HD^+/−^* rats aged 6 months when compared to their control littermates (Figure 4E–H). Interestingly, individual positive PGP 9.5-reactive ORNs were observed in the supporting cell layer in both VNE and MOE. Olfactory marker protein (OMP)-positive cells—a specific marker for mature olfactory and vomeronasal receptor neurons [27,34,35]—showed a more homogenous and ordered cell arrangement for both regions in the 6-month-old animals than the 3-day-old rats (Figure 5A–H). In 3-day-old rats, OMP reactivity in the VNE was mainly detected in the horizontal compartments. The MOE of adolescent transgenic rats showed a significantly higher number of OMP-positive ORNs (Figure 5J).

**Figure 6:**
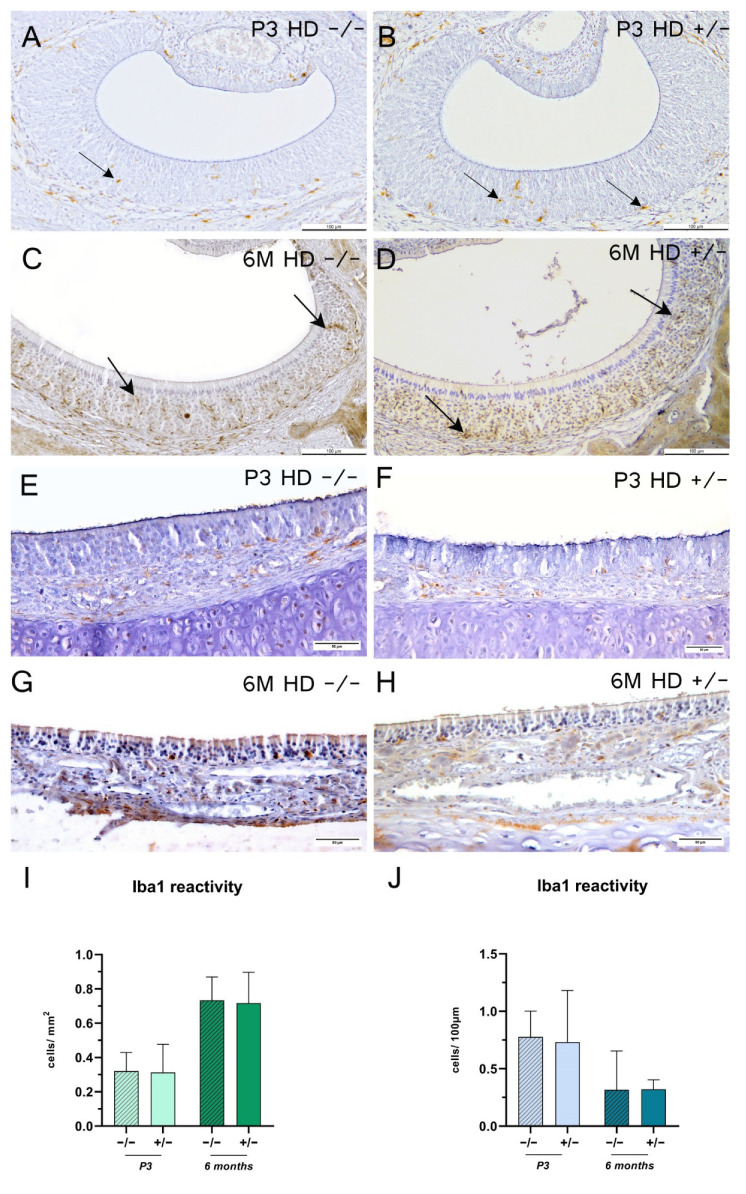
Ionized calcium binding adapter molecule 1 (Iba1) reaction for 3-days-old (P3) and 6-month-old (6 M) rats. (**A**–**H**) Representative immunohistochemical reaction of Iba1- in the vomeronasal epithelium (**A**–**D**) and main olfactory epithelium (**E**–**H**). Arrows indicate Iba1-positive cells. Scale bars: (**A**,**B**) 100 µm; (**C**,**D**) 20 µm; (**E**–**H**) 50µm. Column bars show Iba1-reactive cell counts per mm^2^ (**I**) or per 100 µm (**J**); *n* = 6; (**J**) +/− 6 M: *n* = 5. The significance of differences between groups was tested with mixed-effects analysis followed by Bonferroni’s multiple comparisons (age x genotype interaction: (**I**) *F* value = 0.01017, degree of freedom = 1; (**J**) *F* value = 0.03404, degree of freedom = 1). Data are presented as means ± SD and statistical significance was set at *p* < 0.05.

Finally, reaction with Iba1 antibody, a marker for activated microglia and macrophages [36], showed an overall sparse distribution density for immune cells in the MOE (Figure 6E–H). In both tissues, reaction quantities of Iba1 were genotype-independent. Remarkably, only the VNE revealed an age-dependent, homogeneously distributed increase in Iba1 reactivity (significances not shown).

## 3. Discussion

Early detection of Huntington’s disease (HD) is clinically beneficial for medical and legal affairs, initiation of care plans and lifestyle choices [37]. In this context, olfactory dysfunction might be a prominent early symptom of the disease, as is the case in other neurodegenerative diseases—such as Parkinson’s disease—where hyposmia or anosmia occur years before motor deficits [10,20]. In this study, we investigated, in two different chemosensory regions of the nasal cavity—namely, the main olfactory epithelium (MOE) and the vomeronasal epithelium (VNE)—whether transgenic HD rats reveal an olfactory phenotype with associated disruption of the cellular dynamic. We confirmed that transgenic *HD^+/−^* rats (BACHD line TG22) revealed an extraordinary olfactory phenotype by showing an increase in olfactory receptor neurons (ORNs). On the other hand, we showed that the VNE in BACHD rats emerged as a less sensitive structure that exhibited no cellular changes compared to controls.

### 3.1. Vomeronasal vs. Olfactory Epithelium: The Cellular Homeostasis Is Differentially Affected in BACHD Rats

For the first time, we specifically investigated the peripheral olfactory/vomeronasal systems in HD. We have shown that HD in the two distinct but closely related chemosensory systems of the nasal cavity has measurable effects on the olfactory mucosa and, to a lesser extent, on the vomeronasal system. In our study, we found no changed cellular balance in the VNE with regard to cell proliferation, cell death or neurogenesis in BACHD rats. Cell proliferation (reacted with anti-BrdU) was only present in the lateral zones of the VNE in 6-month-old rats, which is typical for this age [38,39,40]. In contrast, neonatal VNE cell proliferation has been observed in the apical and basal zones [41,42].

In contrast to the VNO, the MOE of 6-month-old BACHD rats displayed a significant increase in OMP-reactive ORNs, with an accompanying increase in PGP 9.5-reactive neurons. PGP 9.5 comprises both mature and differentiating neurons (with the exception of basal cells [43]) whereas OMP neurons are restricted to mature ORNs (Margolis, 1974). The increased number of OMP-positive cells at the 6 month time point could be interpreted as a compensatory measure of the mucosa to balance an early olfactory loss (at 3 months). These data are somewhat unexpected because we had previously demonstrated a substantial reduction in olfactory receptor neurons in the MOE with concomitant disruption of the OE in an animal model of Niemann-Pick type 1C (NPC1) and highly increased compensatory proliferation activity. In addition, the number of apoptotic cells was elevated in NPC1 mice [44]. In the present study, the *HD^+/−^* rats showed neither proliferation nor apoptosis changes, indicating there were no classic signs of degeneration in the MOE. At first glance, it may appear that impaired olfactory performance in a behavior test is at odds with the increased numbers of OMP-positive neurons. However, a closer look at the temporal dynamics of epithelial cell turnover suggests that the findings are not so contradictory. The cell turnover of ORNs is approximately 30 days, and the interval between the behavior test and the cellular analysis was 3 months, enough time for the olfactory mucosa to compensate potential olfactory impairment with increased turnover, at least temporarily [45]. Considering the interdependence between olfactory mucosa and OB [46,47], peripheral olfactory deficits influence the volume and connectivity of the OB and vice versa: Onoda et al. (1988) observed in rabbits that, after bulbectomy, the number of olfactory neurons in the olfactory mucosa initially decreased but then increased and stabilized after 3 months [48]. We therefore speculate that similar central effects may have occurred in our rat model, causing the ORN pool to compensate after 6 months.

### 3.2. Does the BACHD TG22 Line Have an Olfactory Phenotype? Comparison to Other BACHD Lines

In contrast to mouse models (both BACHD and other knock-in constructs [49,50]), increased body weight has not been observed in BACHD rat models [22], which confirms our findings.

Although BACHD rats do not show an early hyperkinetic phase, which is a sensitive predictor for human HD [51], BACHD rats seem to be an appropriate study model for olfaction in HD. The well-investigated lines TG5 and TG9 expressed an olfactory phenotype at the central nervous level (18) that prompted us to focus more closely on peripheral olfaction. Both epithelia in BACHD TG22 rats responded diversely with regard to the cell dynamic. The VNE arises as a conservative structure while the MOE reveals a more variable dynamic, but with no classical degeneration pattern of ORNs. Indeed, the VNE, in contrast to the MOE, has a minor cellular turnover [38]. Weiler et al. [42] postulated that the effective rate of neuronal turnover remains low because newly formed cells are rapidly eliminated after the arrest of adult growth and existing neurons are not replaced. However, in the previously mentioned NPC1 mouse model, we reported an increase in BrdU expression in the VNE of over 200%, in contrast to the hypotheses of Weiler et al. [42] and Barber et al. [38]. Potentially, the different outcomes may result from the fact that different tissues have distinct sensitivities [11,27,44]. What is more, NPC1 is a lipid storage disease that affects virtually all cells and provides a much more dramatic outcome than that seen in discrete HD alterations. The impact of central nervous olfactory impairment in HD has been documented by Lazic et al. (2007) [24] in R6/1 transgenic mice, along with impairment in odor discrimination and identification, but not odor detection, in human patients. Structural abnormalities were additionally observed in the piriform and entorhinal cortices in a mouse model of HD [24]. The reduction in plasticity in the piriform cortex due to aggregate inclusions in huntingtin-affected neurons; however, was disputed by Arrasate et al. (2004), who interestingly observed increased survival rates for neurons that contained such inclusions [52].

Although there was an increase in mature olfactory receptor neurons in the MOE, olfactory detection performance was already diminished in 3-months-old rats (as shown by buried pellet test). This finding may indicate central impairments. Lessard-Beaudoin et al. [23] showed an atrophy of the olfactory bulb in transgenic lines (TG) 5 and 9 of BACHD rats. Regarding mHTT protein concentration in the BACHD rats, the Western blot analysis performed by Yu-Taeger et al. [22] revealed comparable mHTT protein contents in the TG22 and TG9 BACHD rats [23,53]. Moreover, both lines (TG5 and TG9) showed an atrophy in the striatum [22] and Zlebnik et al. [54] described severe deficits in reward motivation without gross motor abnormalities for TG5.

### 3.3. Limitations of the Study

In studying the olfactory phenotype of BACHD rats in TG22, a method of analysis analogous to that used for TG5 and TG9 rats would lead to better comparability, especially in studies of the olfactory bulb. Furthermore, studies of downstream central nervous olfactory structures (e.g., the piriform cortex, insula, orbitofrontal cortices) in this transgenic line are necessary.

In addition, the low biological repetitions for the histological and immunohistochemical reactions and evaluations reduce the reliability and significance of the results. It would also be helpful to conduct a longitudinal study to help interpret odor performance across time. Another concern for this study is the cross-sectional study design, which does not provide a homogeneous dataset. On the other hand, the behavioral experiments tended to reveal high discrepancies and an increase in the repetitions increased the power for statistical evaluation.

## 4. Materials and Methods

### 4.1. Animal Model

For this study, transgenic line 22 (TG22) of the bacterial artificial chromosome Huntington disease (BACHD) rat model was used, which was provided by Prof. Huu Phuc Nguyen from the Institute of Medical Genetics at the University of Tübingen and described by Yu-Taeger et al. [22]. Briefly, the bacterial artificial chromosomes (BACs) were injected into the oocytes of Sprague Dawley rats containing the full-length human huntingtin (*HTT*) gene with 97 CAG/CAA repeats and all regulatory elements. Using the 240 kb RP11-866L6 BAC for the generation of the rat model, the *HTT* exon 1 was replaced with mutant *HTT* exon 1 (*mHTT*) with 97 CAA/CAG trinucleotide repeats and modified through the insertion of two loxP sequence motifs flanking exon 1. Protein expression levels of transgenic mutant huntingtin (mHTT) in various BACHD transgenic lines were described previously, with similar mHTT expression levels in TG22 compared to TG9 but lower than in TG5 [22].

For the experiments, only male rats were considered in order to be concordant with previous experiments [22,55,56,57,58,59]. Transgenic rats (*HD^+/−^* with *mHTT* insertion) or wildtype rats (*HD^-/-^*) aged 3 days or 6 months were used in respective group sizes of six. Genotyping of tail biopsies was performed as previously described by Witt et al. [27]. For the buried pellet test, a cross-sectional approach was chosen to minimize behavioral bias. Therefore, the group size was expanded to ten rats aged 3 months for both male *HD^+/−^* and *HD^−/−^* rats. In order to reduce the number of offspring, male BACHD rats and their wildtype littermates at 8 months of age were used for a representative Western blot analysis (*n* = 1).

Animals were kept in standard cages in a temperature-controlled room (22 ± 2 °C) with a 12/12 h day–night cycle and received ad libitum water and food supply. The study was conducted with the permission of the local Animal Research Committee (Landesamt für Landwirtschaft, Lebensmittelsicherheit und Fischerei (LALLF)) of the state Mecklenburg-Western Pomerania (LALLF M-V/TSD/7221.3-1-047/14) and all rats received humane care according to the EU Directive 2010/63/EU.

### 4.2. Olfaction Detection Ability Test: The Buried Pellet Test

To verify alterations to the olfactory ability, the buried pellet test, following Lehmkuhl et al. [60], as adapted to rats was performed. Before testing, rats had their food restricted for 3 days (food was available for 1 h per day) and thereafter were maintained at about 90% free-feeding body weight during all testing procedures [61]. Two weeks prior to and during food restriction, each tested animal was accustomed to a piece of sweetened cereal pellet, which was later buried (Honey Bsss Loops, Kellogg, Munich, Germany); therefore, each animal received four pieces of the pellets every day. On all six testing days, rats were acclimatized in the testing room for 1 h before the test and were kept in their home cage without a water bottle. For the first five testing days (buried pellet test), freshly cleaned testing cages (Makrolonbox typ IV, UNO BV, Zevenaar, The Netherlands) were prepared with clean ~3 cm bedding, and one pellet was buried 0.5 cm below in one corner of the cage. Importantly, the pellet was buried in a different spot in the cage each day for each trial, and the testing cage and experimenter’s gloves were changed after each rat. For testing, each animal was removed from its home cage and placed in the center of the test cage; the latency time until the rat uncovered the pellet and began eating it was measured. If a rat did not find the pellet within the predetermined time of 300 s, the experiment was terminated, and a latency of 5 min was recorded. Additionally, the experimenter removed the pellet from the bedding, and the rat was allowed to eat it. On testing day 6, the test was repeated using the same scheme, but now the pellet was placed on the surface (surface pellet test). The time it took the rat to find and start eating the pellet was recorded. All trials were video recorded, and the latencies on testing days 1–5 (buried pellet test) and on testing day 6 (surface pellet test) were measured. Due to biological reasons, the 3-day-old rats were not able to perform this test.

### 4.3. BrdU Injections, Weight Measurement and Sample Preparation

**Figure 7:**
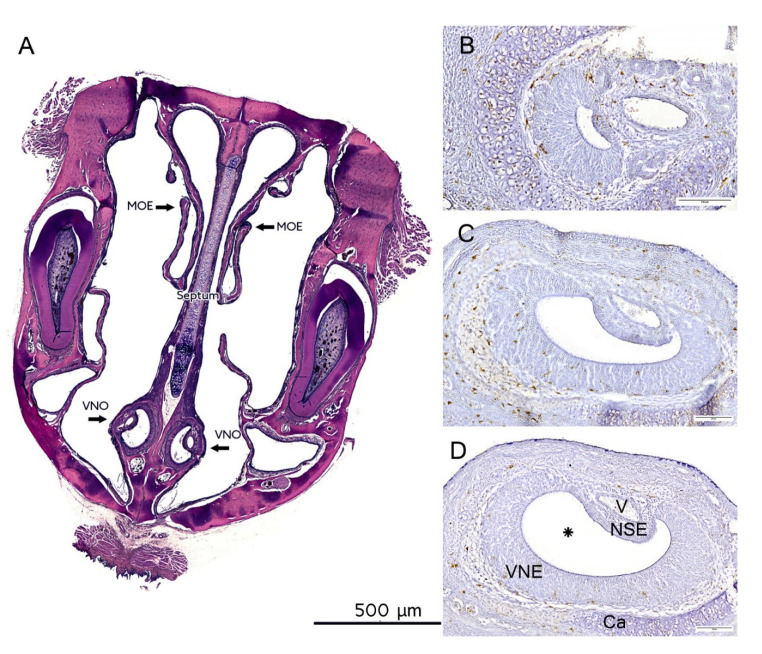
Overview of the vomeronasal organ (VNO) and main olfactory epithelium (MOE) in 3-day-old rats. (**A**) Frontal section across the head of the rat. Samples were subjected to H&E staining. Arrows indicate the MOE and VNO. Scale bar: 500 µm. (**B**–**D**) Representative immunohistochemical reactions with Iba1 in different sectional planes of the VNO. The semilunar-shaped VNO consists of a vomeronasal duct (*), sensory vomeronasal epithelium (VNE), non-sensory epithelium (NSE), vomeronasal cartilage (Ca), vomeronasal nerve and glands (not indicated). V = vein. After Witt and Woźniak [29]. Samples were counter-stained with hematoxylin. Scale bar: 100 µm.

Two hours before euthanasia, rats were i.p. injected with 0.05 mg 5-bromo-2’-deoxyuridine (BrdU; Sigma, St. Louis, MO, USA) per gram of body weight to integrate a thymidine analogue during the S-phase of the DNA synthesis. Therefore, cell proliferation could be immunohistochemically visualized [62,63]. After body weight was measured, the animals were decapitated and the skull was dissected. The specimens were fixed in 3.7% paraformaldehyde (PFA, pH 7.0 (Merck, Darmstadt, Germany) in 0.1 M PBS, pH 7.4 (Carl Roth, Karlsruhe, Germany)), for one week and transferred into 1% ethylenediaminetetraacetate solution (EDTA; Sigma-Aldrich, Darmstadt, Germany) for decalcification at 37 °C for up to two weeks. Six-month-old rats were euthanized with an overdose of anesthesia (xylazine (Bayer Animal Health, Leverkusen, Germany) and ketamine (Bela-Pharm, Vechta, Germany)) and perfused with ice-cold 0.9% NaCl (Carl Roth, Karlsruhe, Germany) and 3.7% PFA. After skull dissection, specimens were post-fixed for three to four days in 3.7% PFA and decalcified in 1% EDTA solution at 37 °C for up to one month. Finally, specimens were embedded in paraffin using standard protocols and serially cut in 10 µm thick frontal sections to reveal the main olfactory epithelium (MOE) and the vomeronasal organ in the ventral nasal septum (VNO, Figure 7A). For the representative Western blot analysis, the brain was dissected and separated into the olfactory bulb, cerebellum, brain stem, hypothalamus, hippocampus, cortex and striatum. Afterwards, brain tissue was snap frozen with liquid nitrogen and stored at −80 °C.

### 4.4. Western Blot Analysis

Sample preparation and Western blot analysis were performed following Gray et al. [50]. Anti-huntingtin protein MAB2166 (Sigma-Aldrich, St. Louis, MO, USA) was diluted 1:1000. For a detailed description, see Supplementary Materials Figure S2.

### 4.5. Sample Selection

In order to quantify the blind-ending bilateral vomeronasal epithelium (VNE), the starting point for the subsequent immunohistochemical reaction was set once small volumes of sensory epithelium were already present (compare Figure 7B). In postnatal rats, a distance of 250 µm was selected between the sections, while in the adult animals, a distance of 750 µm was specified between the sections owing to the larger volume, resulting in *n* = 7 sectional planes per individual. In order to analyze the VNE semi-quantitatively, a sectional plane in the lower area (Figure 6B), one in the middle area (Figure 6C) and one in the upper area (Figure 6D) were selected, resulting in a total number of *n* = 3 per individual. MOE sections were selected according to the VNE sections.

### 4.6. Histology and Immunohistochemistry

A detailed description of the immunohistochemical procedures is provided in Supplementary Materials Figure S1. Hematoxylin (Merck, Darmstadt, Germany) and eosin (Carl Roth, Karlsruhe, Germany; H&E) staining was performed every 100 µm for routine inspection after standard protocols. For the assessment of cell proliferation, cell apoptosis, olfactory neuron cells, immature and mature neurons and gliosis, immunohistochemistry was performed with antibodies directed against BrdU [35,64], activated caspase-3 [31,32], protein gene product 9.5 (PGP 9.5) [33], olfactory marker protein (OMP) [65] and ionized calcium binding adapter molecule 1 (Iba1) [36]. For technical issues, the activated caspase-3 reaction was performed only in 6-month-old rats. After deparaffination and antigen retrieval in a microwave in citrate buffer, endogenous peroxidases were blocked using 3% H_2_O_2_ solution in 0.1 M PBS. Then, slides were exposed to rat anti-BrdU (1:1000; AbD Serotec, Oxford, UK), rabbit anti-activated caspase-3 (1:500; Cell Signaling Technology, Cambridge, UK), rabbit anti-OMP (1:6000; Sigma-Aldrich, St. Louis, MO, USA), rabbit anti-PGP 9.5 (1:2000; Millipore, Darmstadt, Germany) or rabbit anti-Iba1 (1:1000; Wako Pure Chemical Industries, Neuss, Germany) antibodies. After overnight incubation at 4 °C, secondary antibodies were incubated for one hour at room temperature (biotinylated anti-rabbit or anti-rat; Vector Laboratories, Biozol Diagnostica Vertrieb GmbH, Eching, Germany), followed by one-hour incubation with an avidin/biotin-based amplification kit (Vectastain Elite, Biozol Diagnostica Vertrieb GmbH, Eching, Germany). Visualization was performed with 3,3’-diaminobenzidine tetrahydrochloride, followed by optional counter-staining with hematoxylin (depending on cell density), dehydration, mounting in DePeX (Serva Electrophoresis GmbH, Heidelberg, Germany) and coverslipping.

### 4.7. Quantification and Statistical Analysis

Images were captured with a Leica DM6 B microscope (Leica Microsystems CMS GmbH, Wetzlar, Germany) equipped with a DMC6200 camera. For the VNE, the area of the sensory epithelium (= the region of interest) was measured and positive-reacted cells counted manually in ImageJ (version 1.53p, Bethesda, MD, USA), whereas for PGP 9.5 and OMP, positive cells were semi-automatically quantified with ImageJ (protocol provided in Supplementary Materials S3), resulting in a ratio of positive cells to mm^2^. PGP 9.5 in the VNE was not analyzed in 3-day-old animals for technical reasons. Samples for manual recording were counted in a blinded fashion and, despite the fact of blinding, the morphological structures of the 3-day-old and 6-month-old rats were distinctly evident.

For the MOE, the distance to the olfactory epithelium was measured, and positive-reacted cells were counted manually in ImageJ, forming a ratio of positive cells to 100 µm.

Statistical analysis was performed with GraphPad Prism 9.2.0 (GraphPad Software Inc., San Diego, CA, USA) and data are represented in boxplots (for body weight) or column bar graphs (for staining results) as mean values ± SD. Data were checked for normality with a Shapiro–Wilk test. In order to compare the genotype and age interaction, mixed-model effects analysis was performed followed by Bonferroni’s post hoc test for multiple comparison of means. If only the genotype was compared, an unpaired two-sided t-test was performed. For more details, see the respective figure legends.

## 5. Conclusions

We characterize for the first time the cell dynamics of two closely related chemosensory organs, the VNE and MOE, in BACHD rats and suggest that the question of whether BACHD rats exhibit an olfactory phenotype can be answered positively. This offers the possibility of furthering investigating the molecular nature of olfactory deficits in HD.

## Figures and Tables

**Table 1:** Activated caspase-3-positive cells in the vomeronasal epithelium (VNE) and in the main olfactory epithelium (MOE) in 6-month-old animals.

	VNE		MOE	
Genotype	+/−	−/−	+/−	−/−
Animal number (*n*)	6	6	6	5
Mean	24.67	32.50	1.40 × 10^−5^	1.25 × 10^−5^
Standard deviation	11.66	29.67	7.98 × 10^−6^	6.27 × 10^−6^
Test performed	Mann–Whitney test		Unpaired t-test (two-tailed)	
Significance level	ns		ns	

## Data Availability

The data are available on request from the corresponding author.

## Supplementary Materials

The following supporting information can be downloaded at: https://www.mdpi.com/article/10.3390/ijms23147625/s1.
