# Supplementary material for: Differential Cellular Balance of Olfactory and Vomeronasal Epithelia in a Transgenic BACHD Rat Model of Huntington’s Disease
Source: Int J Mol Sci. 2022 Jul 10;23(14):7625. doi: 10.3390/ijms23147625 (PMC9316117; doi:10.3390/ijms23147625)
Supplement: Supplementary file 1 [file ijms-23-07625-s001.zip › ijms-1763450-supplementary.pdf]

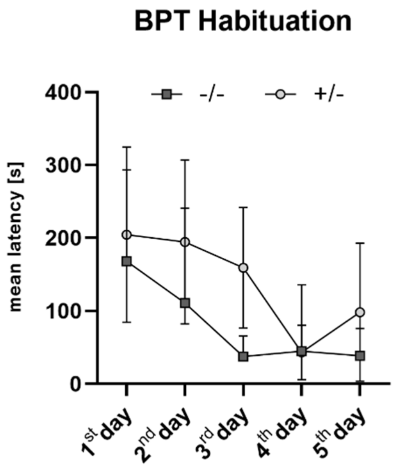

Figure S1: Habituation task prior to the buried pellet test (BPT). Mean latency times in the habituation test of 3-months-old rats. No significant differences were found between the genotypes.

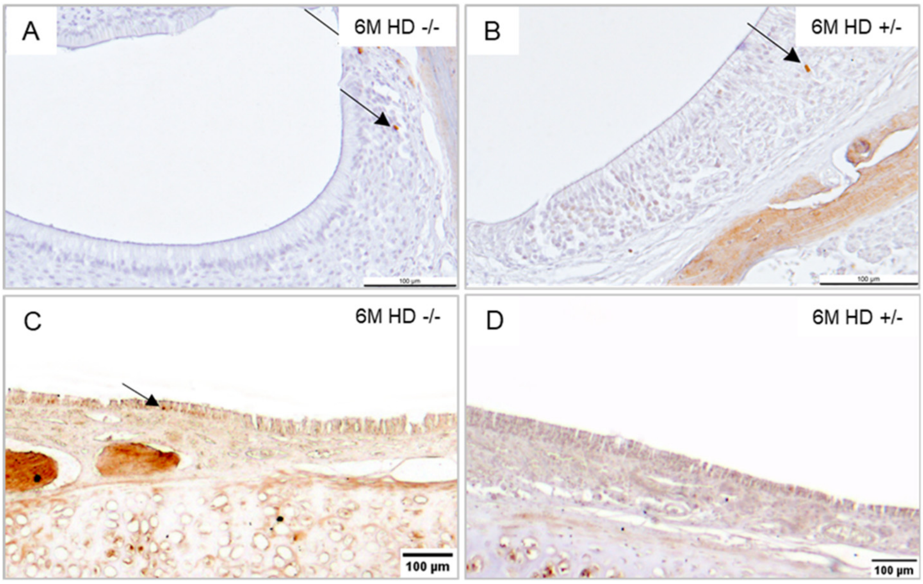

Figure S2: Active caspase-3 reaction of 3-days old (P3) and 6 –month (6M)-old rats. Immunohistochemical reaction of caspase-3-positive cells in the vomeronasal epithelium (A-D) and main olfactory epithelium (E-H). Arrows indicate caspase-3- positive cells. Scale bars: A-D: 100µm; E-H: 50µm.

S3 Staining protocols

Immunhistochemical reactions

1. Deparaffination 3x for 10 min in xylene followed by descending ethanol series (100 %, 96 %, 90 %, 80 %, 70 %) until distilled water for 1 min, respectively
2. Antigen retrieval in citrate buffer in the microwave according subsequent table S1. Afterwards, samples in the sample holder were cooled down with sourrounded ice

Table S3: antibody dilutions and pretreatment times

Age	Primary antibody	Dilution	Secondary antibody	Antigen retrievel in microwave
**P3**	OMP (rabbit)	1 : 6000	Biotinylated Anti-Rabbit 1 : 200	5 min at 850 W + 5 min at 340 W
**P3**	BrdU (rat)	1 : 1000	Biotinylated Anti-Rat 1 : 200	5 Min at 680 W
**P3**	PGP 9.5 (rabbit)	1 : 1000	Biotinylated Anti-Rabbit 1 : 200	5 min at 850 W + 5 min at 340 W
**P3**	Iba1 (rat)	1 : 1000	Biotinylated Anti-Rabbit 1 : 200	2 min at 850 W
**6Mo**	OMP (rabbit)	1 : 6000	Biotinylated Anti-Rabbit 1 : 200	5 min at 850 W + 5 min at 340 W
**6Mo**	BrdU (rat)	1 : 2000	Biotinylated Anti-Rat 1 : 200	5 min at 680 W
**6Mo**	activated caspase-3 (rabbit)	1 : 500	Biotinylated Anti-Rabbit 1 : 200	5 min at 680 W
**6Mo**	PGP 9.5 (rabbit)	1 : 1000	Biotinylated Anti-Rabbit 1 : 200	5 min at 850 W + 5 min at 340 W
**6Mo**	Iba1 (rabbit)	1 : 1000	Biotinylated Anti-Rabbit 1 : 200	2 min at 850 W

3. Peroxidase inhibition with 3 % H_2O_2 in a wet chamber for 30 min at room temperature
4. Blocking of the non-specific binding sites with PBS, normal goat serum (approx. 5 %) and 0.05 % Triton in a wet chamber for 1 h at room temperature
5. Short washing with 0.1 M PBS
6. According to table S1, primary antibodies were diluted in 0.1 M PBS and 3 % normal goat serum. Incubation in a wet chamber overnight at 4°C
7. 3x washing step with 0.1 M PBS for 5 min
8. Secondary antibodies were diluted 1 : 200 in 0.1 M PBS, 3 % normal goat, and 0,05 % triton and incubated in a wet chamber for 1 h at room temperature
9. Component A and component B from the avidin/biotin (AB-)complex by Vectastain was diluted 1:50 in 0.1 M PBS and incubated at least 30 min prior to use
10. 3x washing step with 0.1 M PBS for 5 min
11. Incubation of the AB-complex in a wet chamber for 1 h at room temperature
12. 3x washing step with 0.1 M PBS for 5 min
13. Preparation of DAB solution 1 : 50 in 0.1 M PBS with 1 : 10 000 diluted 35 % H_2O_2
14. Incubation of DAB for 4 min at room temperature
15. 3x washing step with 0.1 M PBS for 5 min

16. Optional: Counterstaining with hemalaun for < 1 s and stopping the reaction with tap water
17. Dehydration with increasing propanol series (70 %, 80 %, 90 %, 96 %, 100 %) for 1 min and 3x for 10 min in xylene
18. Mounting in DePeX and coverslipping

S4 Image J code

Prior to use this code, VNE regions were cut manually. For other images, Contrast and threshold ranges may vary.

OMP macro:

```
run("Set Scale...", "distance=341.0015 known=100 unit=um global");
run("8-bit");
run("Brightness/Contrast...");
setMinAndMax(73,238);
run("Apply LUT");
run("Enhance Contrast...", "saturated=0.4 normalize");
run("Sharpen");
run("Threshold...");
setAutoThreshold("MaxEntropy");
setThreshold(1, 97);
setOption("BlackBackground", false);
run("Convert to Mask");
run("Close");
run("Fill Holes");
run("Remove Outliers...", "radius=2 threshold=50 which=Dark");
run("Analyze Particles...", "size=2-500 show=[Overlay Masks] display clear summarize add slice");
roiManager("Show All with labels");
roiManager("Show All");
roiManager("Save", "");
run("Summarize");
close();
```

PGP macro:

```
run("Set Scale...", "distance=341.0015 known=100 unit=um global");
run("8-bit");
run("Brightness/Contrast...");
setMinAndMax(78, 167);
run("Apply LUT");
run("Enhance Contrast...", "saturated=0.4 normalize");
```

```
run("Sharpen");
run("Minimum...", "radius=1");
run("Threshold...");
setAutoThreshold("Triangle");
setThreshold(0, 75);
setOption("BlackBackground", false);
run("Convert to Mask");
run("Close");
run("Remove Outliers...", "radius=4 threshold=50 which=Dark");
run("Watershed");
run("Analyze Particles...", "size=3-1000 show=[Overlay Masks] display clear summarize add composite");
roiManager("Show All with labels");
roiManager("Show All");
roiManager("Save", "");
run("Summarize");
close();
```
